# Modulating Reaction Kinetics Using an Electrolytic Method to Achieve Efficient Vehicle Identification Number Reappearance

**DOI:** 10.3390/mi16050578

**Published:** 2025-05-15

**Authors:** Jintao Wang, Xiaoshun Zhang, Mengfan Chen, Xihao Zhang, Zhongliang Zhang, Jianguo Liu

**Affiliations:** 1 School of Forensic Science, Criminal Investigation Police University of China, Shenyang 116049, China; jintaowang26@outlook.com (J.W.); zhongliangzhang11@outlook.com (Z.Z.); 2 Key Laboratory of Impression Evidence Examination and Identification Technology, Ministry of Public Security, Shenyang 110035, China; 3 School of Chemistry and Life Science, Anshan Normal University, Anshan 114007, China; mengfanchen0601@outlook.com; 4 Institute of Metal Research, Chinese Academy of Sciences, Shenyang 110016, China; xhzhang19s@imr.ac.cn (X.Z.); jgliu@imr.ac.cn (J.L.)

**Keywords:** Q235 steel corrosion, corrosion inhibitor, electrochemical etching, reaction kinetics, forensic science, vehicle identification number

## Abstract

Vehicle identification number (VIN) reappearance technology is an important means of vehicle traceability in various criminal cases. However, with the advancement of metallurgical techniques, the corrosion resistance of metal becomes stronger, and the traditional chemical etching reappearance method gradually fails. In order to break through the dilemma of traditional methods, this study establishes an electrochemical corrosion system by introducing the corrosion inhibitor hexamethylenetetramine (HMTA) to precisely regulate the electrochemical dissolution kinetics. Material characterization and electrochemical measurements demonstrated that the selective adsorption of HMTA significantly enhances the potential difference between plastically deformed regions and the normal metal substrate (ΔE_max_ = 6 mV). By effectively suppressing the corrosion rate in non-target areas, HMTA promotes selective anodic oxidation reactions in the vehicle identification number character regions due to their distinct microstructural characteristics, thereby substantially improving the contrast of the reappeared VIN markings. Density functional theory calculations and molecular dynamics simulations further reveal the formation of a dense adsorption layer, which is a key factor in improving the reproducibility of the results. The experimental results demonstrate that under conditions of 6 V applied voltage, with 0.5 M hydrochloric acid and 0.02–0.03 M HMTA in the electrolyte, efficient VIN reappearance could be achieved within 3–4 min on filed-down surfaces.

## 1. Introduction

With the development of steel industries and automobiles, the global motorized vehicle ownership has increased rapidly [1,2,3]. However, while automobiles bring great convenience to society, they also trigger a series of negative problems [4,5,6]. Due to the flexibility and covert nature of motor vehicles, they have gradually become diversified crime carriers [7,8]. In theft, the smuggling of motor vehicles, terrorist attacks using motor vehicles, and related crimes, criminals usually tamper with or grind the vehicle identification number that represents the identity information of the motor vehicle to conceal their identities and evade legal sanctions [9,10]. The vehicle identification number (VIN) is a unique identification number of motor vehicles, carrying a wealth of original vehicle information. By enquiring about the VIN, a great amount of important information can be obtained from the production registration of a motor vehicle, as it plays a significant part in the identification of the vehicle ownership, which is similar to deoxyribonucleic acid (DNA). In view of the irreplaceable role of VIN inspection technology in cases involving motor vehicles, it has received extensive attention from forensic science practitioners worldwide.

Owing to the continuous development of forensic science, a series of inspection methods such as chemical etching, magnetic particle inspection, electron backscatter diffraction, and infrared thermography have been developed [11,12,13]. Among them, magnetic particle inspection has received widespread attention due to its advantage of non-destructive testing, which mainly uses the differences in magnetic parameters such as coercivity and permeability between the stamped lettering part and the metal substrate to achieve the reproduction of the VIN. However, its practical application involves precision instruments such as Hall sensors, magnetoresistive sensors, and eddy current magnetometers, which have been gradually eliminated considering the cost and operational difficulties [14]. After continuous practical screening, the chemical etching method became the main method of VIN reappearance due to its simple and efficient characteristics, which only relies on the cotton sleeve dipped in the configured corrosion solution for application in order to display the VIN. However, with the continuous progress of steel forging technology and the improvement in corrosion resistance, the traditional chemical etching method has gradually failed to meet the current needs [15,16]. To solve the problem of difficult VIN recovery, an electrochemical etching method has been proposed to increase the corrosion rate by applying an external current [17,18]. Limited by the lack of systematic scientific experimental studies and the absence of testing parameter standards, it is difficult to provide scientific guidance for practical applications [19,20]. In addition, it has been proven in practice that electrochemical methods have problems, such as corrosion rates that are too fast and an uncontrollable etching process, which often lead to failure due to excessive etching. Therefore, the development of a method that can accurately regulate the kinetic rate of the electrolysis reaction has become a key problem to be solved in this field.

Hexamethylenetetramine (HMTA) is a hybrid surfactant with a unique cage-like structure consisting of four mutually thickened triazacyclohexane rings. This special structure endows HMTA with good thermal stability, strong adsorption capacity, and wide applicability, which makes it widely used in many fields such as pharmaceuticals, chemicals, and materials [21,22]. In the field of corrosion prevention and control, HMTA, as a commonly used corrosion inhibitor, is able to effectively inhibit the erosion of corrosive media on metal substrates at the molecular level. At the same time, benefiting from its good thermal stability, HMTA can maintain structural stability and continue to play a protective role even under the high-temperature environments generated by the current during electrolysis. More critically, this inhibition provides new ideas and possibilities for regulating the reaction rate during the electrochemical etching process.

Herein, HMTA was innovatively introduced as a corrosion inhibitor to optimize the electrochemical etching method, which successfully solved the problems of fast corrosion rates and poor contrast of reproducible results for the traditional electrochemical etching method. Microscopic characterization of the treated Q235 metal surface by scanning electron microscopy (SEM) and atomic force microscopy (AFM) visually confirms the significant effect of HMTA on the metal surface morphology. Fourier-transform infrared spectroscopy (FTIR) and X-ray photoelectron spectroscopy (XPS) analyses further revealed that the N atoms in the corrosion inhibitor are chemically bonded to the Fe atoms of the metal, forming a stable adsorbent layer on the metal surface, which effectively blocks the attack of aggressive particles. The electrochemical characterization results show that the potential difference between the plastic deformation zone and the normal metal substrate is significantly increased due to the presence of the HMTA adsorption layer, which greatly enhances the contrast of the VIN reproduction results and improves the clarity and accuracy of the reproduction. Finally, the regulatory mechanism of HMTA molecules forming coordination bonds with iron atoms to cover the active sites on the Q235 surface is thoroughly investigated at the microscopic level by molecular dynamics (MD) simulations and density functional theory (DFT) calculations. Theoretical calculations show that this mechanism can effectively reduce the attack of aggressive particles, such as hydrogen and chloride ions, on the metal surface and accurately regulate the reaction rate during the electrolysis process.

The results of this study show that the introduction of HMTA provides a practical and effective solution to the problem of VIN reproduction in electrochemical etching. This technique not only achieves a breakthrough in technology, but also has the advantages of easy operation, controllable cost, and high reproduction accuracy, which shows great potential for application in the fields of the judicial system, crime investigation, and vehicle management, and is expected to become the development direction of VIN reappearance technology in the future.

## 2. Materials and Methods

### 2.1. Chemicals and Materials

The Q235 steel was purchased from Xinghua Ruishun Electromechanical Equipment Co., Ltd. (Xinghua, China), with the following composition: 96.7 wt.% Fe; 3.05 wt.% O; 0.15 wt.% Si; 0.07 wt.% Mg; and 0.04 wt.% Ca. Hexamethylenetetramine was obtained from Harbin Ruibiao Technology Co., Ltd. (Harbin, China); ethanol (C_2_H_5_OH), hydrochloric acid (HCl), and nitric acid (HNO_3_) were all obtained from China National Pharmaceutical Group Corporation (Beijing, China). All reagents employed in the experiment were of analytical grade and could be utilized without the necessity for further purification. The DC power supply was acquired from Shenzhen Solid Test Electronic Technology Co., Ltd. (WPS305H, Shenzhen, China). Degreased cotton sleeves were bought from Xingchi Gilding Equipment Factory (Liaocheng, China). The grinding machine was purchased from Shanghai Meinait Industrial Co., Ltd. (MNT070332, Shanghai, China).

### 2.2. Sample Preparation

The Q235 steel plates were sliced into steel sheets with dimensions of 2 cm in length and width and a depth of 0.18 cm. Characters were stamped using a punching tool, and the steel sheets were fixed into plastic molds embedded in PVC pipes, ensuring that the steel sheets remained vertically fixed while being able to move up and down. A cylindrical steel hammer, which had a dimension less than the internal caliber of the PVC pipe, was dropped from a fixed height, and a clear, uniform character was engraved on a carbon steel plate by the impact of free fall. Then, the engraved characters were treated to simulate the method used by criminals to erase vehicle identification codes. A grinding machine equipped with a 90-mesh grinding bit was used at a speed of level 5 for five minutes to evenly grind the steel plates, resulting in carbon steel plate samples where the engraved characters were no longer identifiable.

### 2.3. Reappearance of Engraved Marks

**Figure 1:**
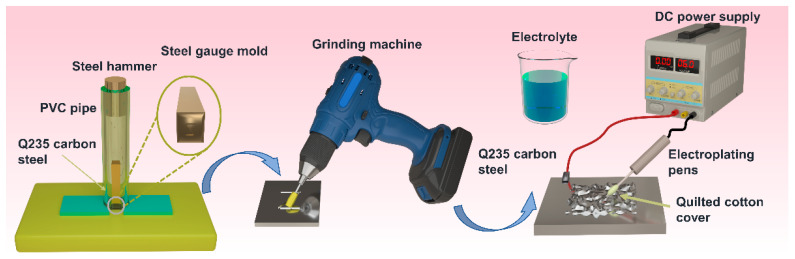
Schematic of vehicle identification number (VIN) display process via electrolytic method.

The electrolyte solution was prepared by mixing 0.5 M HCl with HMTA at concentrations of 0 M, 0.01 M, 0.02 M, 0.03 M, and 0.04 M, respectively. The specific steps of the electrochemical visualization experiment are as follows in Figure 1. Firstly, a wire with an alligator clip at one end was used to connect the Q235 sample to the anode of the DC power supply, and an electroplating pen with a cotton sleeve was linked to the cathode of the power supply. Then, the electroplating pen was dipped into the electrolyte in a beaker, the power supply was turned on, and the voltage was adjusted to 6 V. After that, the electroplating pen was repeatedly wiped and applied over the potential stamped character area on the steel sheet to perform electrolytic corrosion. During the reaction, the electrolyte was reapplied at 1 min intervals to prevent the danger of short-circuiting due to insufficient electrolyte. The visualized results were photographed and recorded in a timely manner to avoid over-corrosion that could affect the clarity of the characters. HCl and HMTA (C_6_H_12_N_4_) were, respectively, designated as H and C.

### 2.4. Surface Analysis

The electrochemical reaction was conducted on Q235 carbon steel using an electrolytic method, followed by surface morphology analysis of the original and corroded surfaces through a scanning electron microscope (SEM) (JEOL, JSM-6300, Tokyo, Japan). The deformed cross-sections of the metal sample were burnished with #240-#6000 waterproof sandpaper, and after treatment with metallographic reagent (4% HNO_3_ and C_2_H_5_OH), the cross-section morphology was observed under a metallographic microscope (OM, Observer. Z1m, ZEISS, Jena, Germany). A contact angle tester was used to compare the coarseness of carbon steel after being corroded in two different electrolytes (JC2000C1, Chengde Jinhao Instrument Manufacturing Co., Ltd., Chengde, China) and an atomic force microscope (AFM) (Bruker Dimension Icon, Karlsruhe, Germany). After immersing in 0.5 M HCl and 0.5 M HCl + 0.02 M HMTA for 6 h, the elemental composition, functional groups, and chemical state changes on the surfaces of the carbon steel were surveyed using X-ray photoelectron spectroscopy (XPS) (Thermo VG, EscaLab 250, Waltham, MA, USA) and Fourier-transform infrared spectroscopy (FTIR) (Thermo Fisher Scientific Nicolet iS20, Waltham, MA, USA).

### 2.5. Electrochemical Characterization

The electrochemical impedance spectra (EIS) and polarization curves for the two electrolytes were measured using a typical three-electrode workstation (Gamry, Reference 3000, Warminster, PA, USA). The reference electrode was a saturated calomel electrode (SCE), the counter electrode was a platinum sheet, and the working electrode was the treated Q235 steel sheet. Prior to testing, the Q235 steel was burnished to a smooth mirror finish using #240-#2000 sanding paper. Specifically, electrochemical tests were performed in a solution with 0.5 M H and 0.02 M C. Linear polarization measurements were carried out over a range of ±20 mV with respect to the open circuit potential (OCP) at a scan rate of 0.166 mV s^−1^. Electrochemical impedance measurements were carried out at the OCP over a frequency range of 0.1 Hz to 100,000 Hz, with a perturbation signal amplitude of 10 mV. Charge-transfer resistances (*R_ct_*) and double-layer capacitances (*C_dl_*) were derived from the Nyquist diagrams; relevant data were fitted to the corresponding impedance values and other parameters using an equivalent circuit through ZView software 2.4.

### 2.6. Computational Details

To analyze the interaction between HMTA and Q235 substrate, density function theory (DFT) and molecular dynamics (MD) simulations were performed using Gaussian 16 and CASTEP 19.1 linux packages, respectively. In DFT calculations, the structure optimization and single-point energy calculation were performed at the B3LYP-D3 (BJ)/6-311G (d,p) level. In MD simulations, molecular models were constructed through the amorphous cell module, and the chemisorption of HMTA molecules on the Q235 steel (110) surface was carried out via the Forcite module. The Forcite module was used to calculate the equilibrium configurations of polymer chains and their absorption energies. The 15 Å vacuum layer was selected to ensure duplicate plate decoupling. The positions of all iron atoms were determined. In total, 1000 water molecules, 75 HCl molecules, and 30 HMTA molecules were selected for the study. The computational details were chosen as follows: dynamics is used as a computational task, the system synthesis is NVT, and the total simulation time is 5000 ps. The Berendsen barostat and Andersen thermostat were selected for pressure and temperature control, respectively.

## 3. Results and Discussion

**Figure 2:**
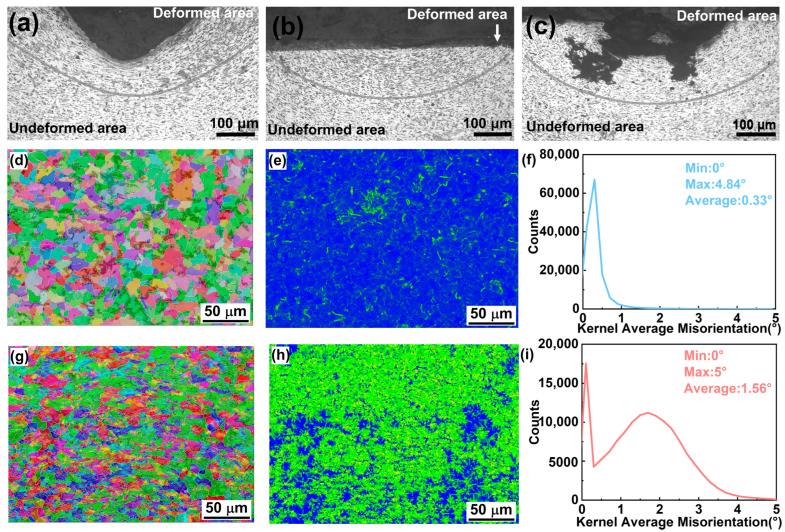
Cross-sectional morphology of carbon steel (**a**) after stamping, (**b**) after the grinding, and (**c**) after electrolytic manifestation; IPF plots (**d**), KAM plots (**e**), and KAM curves (**f**) for EBSD testing of undeformed carbon steel; and IPF plots (**g**), KAM plots (**h**), and KAM curves (**i**) for EBSD testing of stamped deformed carbon steel.

Firstly, metallographic microscopy and EBSD tests were used to analyze the changes in the lattice structure of the metal. The metallurgical microscope observation shows that the structural difference in the die area of the Q235 stamped section in Figure 2a is obvious, and the plastic deformation at the bottom elongated the grains. Figure 2b shows that even if the depression on the surface of the stamped mark is removed by polishing, the internal deformation area still exists; because of this, the part of the potential stamped mark shows a depression trace after electrochemical etching (Figure 2c). Further EBSD tests also matched the metallurgical microscopy results. Comparing the two EBSD images, the crystal orientation in Figure 2d shows a diffuse distribution, regular grain morphology, and smooth and continuous grain boundaries, indicating a stable material microstructure. Figure 2g has obvious aggregation of crystal orientation, strong weaving, and significant differences in grain morphology and size, indicating that it undergoes intense plastic deformation. In addition, compared with Figure 2e, which has a relatively homogeneous and uniform color distribution, Figure 2h has a rich and complex distribution of colors, which indicates that the crystal orientation in the deformation zone is more diverse. The KAM value of the undeformed sample (Figure 2f) ranges from 0° to 4.84°, with an average value of 0.33°. The peaks of the curves are obvious and concentrated in the region of low KAM values. This indicates that most of the regions within the material have small orientation differences between neighboring grains, low lattice distortion, and low microscopic stresses within the material. The KAM values of the deformation samples (Figure 2i) range from 0° to 5°, with an average value of 1.56°. The curve is relatively flat, and the peak location is biased towards the higher KAM value region. This indicates that the orientation difference between neighboring grains is more widely distributed within the material, reflecting the existence of large, microscopic stresses within the material. These results indicate that the plastic deformation induced by stamping significantly alters the microscopic stress distribution within the metal, resulting in a corrosion potential difference between the deformed and undeformed areas of the metal, which provides the possibility of the reappearance of the VIN.

**Figure 3:**
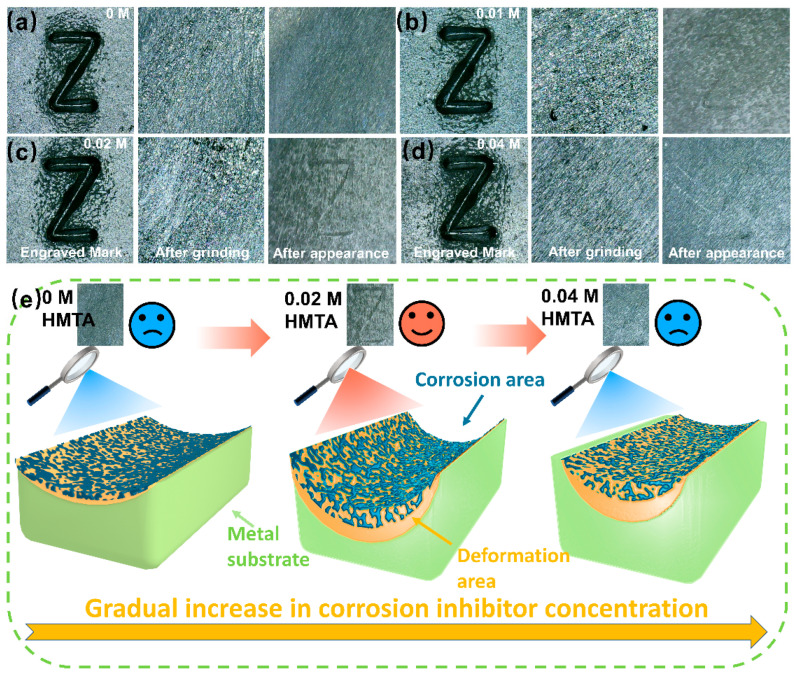
Electrolysis results at 6 V, 0.5 M HCl, and various hexamethylenetetramine (HMTA) concentrations of (**a**) 0 M, (**b**) 0.01 M, (**c**) 0.02 M, and (**d**) 0.04 M; (**e**) schematic diagrams of manifestation results of characters.

After completing the characterization and analysis of the VIN reproduction mechanism, related experiments were carried out. Although the electrochemical manifestation method improves the poor corrosion effect and slow imaging speed of the traditional chemical imaging method by applying electric current, it suffers from the disadvantages of poor reproduction effect and excessive corrosiveness of the hydrochloric acid electrolyte. In view of this, hexamethylenetetramine is introduced in this study to regulate the reaction rate of the method to optimize the electrochemical imaging process. After a series of experiments as displayed in Figures S1–S15 and Tables S1–S15, the preliminary conditions of 6 V voltage and 0.5 M HCl concentration are chosen based on a comprehensive consideration of safety, repeatability, and time required. Based on this, we further analyze the reproducibility under different concentrations of HMTA, and the research outcomes are presented in Figure 3a–d and Table 1. When the concentration of HMTA is 0.01 M, the corrosion inhibition effect is poor, failing to provide sufficient protection for the carbon steel samples. As the concentration of HMTA increased to 0.02 M and 0.03 M, the reproduced VIN markings were clear and had strong contrast, as observed in Figure 3b and Figure S16. At a concentration of 0.04 M, insufficient etching strength increased the required reappearance time, and it caused rapid heating of Q235 with a risk of short circuits. Figure 3e illustrates the VIN manifestation results from the perspective of a character cross-section. At a low concentration, a poor effect is observed where, although the etching intensity is high, the discrepancy in corrosion depth between the plastically deformable region and the normal metal substrate is small. With the increase in HMTA, the contrast depth of Q235 is increased after etching, and the display effect is noticeably improved, but along with that, the electrochemical etching ability weakens, and the clarity of reproduced characters seriously declines.

**Figure 4:**
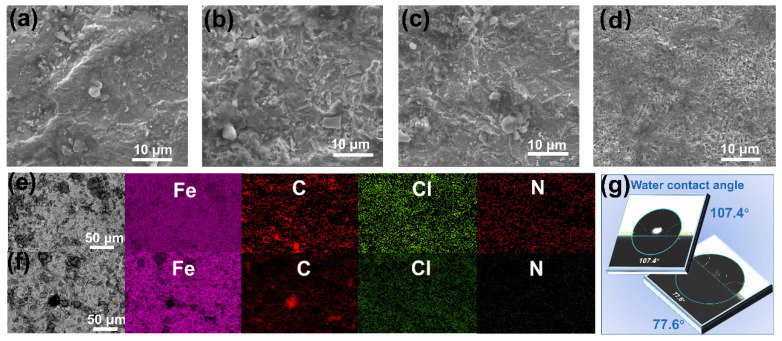
SEM morphology of (**a**) deformed carbon steel and (**b**) undeformed Q235 after 6 h immersion in 0.5 M H electrolyte; SEM morphology of (**c**) deformed carbon steel and (**d**) undeformed carbon steel after 6 h immersion in 0.5 M H + 0.02 C electrolyte; EDS spectra of Q235 after 6 h immersion (**e**) in 0.5 M H electrolyte and (**f**) in 0.5 M H + 0.02 C electrolyte; and (**g**) water contact angle.

For the purpose of investigating the corrosion inhibition mechanism of HMTA, various characterizations were carried out in this study. The influence of various proportions of HMTA on the surface morphology of Q235 is observed by SEM. Figure 4a,b show the SEM morphology of the deformed region (the stamped character area) and the undeformed region (the metal matrix) after electrochemical etching in a 0.5 M H electrolyte, respectively. It can be seen that both the deformed and undeformed regions suffered severe corrosion, and more corrosion products occur in the deformed region than undeformed region. This is due to the fact that the potential difference makes the deformed region more reactive and the electrochemical corrosion etching more severe. Figure 4c,d demonstrate the morphology of the deformed and undeformed regions after treatment with 0.02 M HMTA. Obviously, the corrosion products were significantly reduced in both regions, and the degree of corrosion was alleviated. This demonstrates that HMTA plays a buffering role in the electrochemical etching process for character revelation under high-voltage and high-current conditions [23,24]. The introduction of HMTA remarkably controlled the corrosion rate during the electrochemical etching process to reveal the potential stamped characters on the metal [25,26,27]. From the above SEM results, the comparison between the plastically deformable area and the normal metal is clearly obvious before and after HMTA is added, and this difference, caused by the different etching rates, is the main reason for the reappearance of the VIN.

According to the EDS analysis (Figure 4e,f), on the surface of Q235 steel without the use of HMTA, the Cl element is distributed relatively uniformly, and the Fe content is high. This is because the surface of Q235 steel has undergone severe corrosion, resulting in the formation of a large number of porous corrosion products on the Q235 surface. The microporous structure of these products allows Cl^−^ to freely penetrate and accumulate at active sites such as grain boundaries. For the samples treated with the electrolyte containing HMTA, the C content increased, the Fe content decreased, and the distribution of Cl became uneven, along with some localized concentrations. This may be due to the sorption of HMTA molecules on the metal surface and the formation of a shielding film that prevents the uniform distribution of Cl^−^. Furthermore, the appearance of N elements, which are absent in the sample treated without additives, further indicates the successful adsorption of HMTA molecules on the metal surface [28,29]. The contact angle of the sample addressed in the 0.5 M H + 0.2 M C electrolyte is 107.4° according to the contact angle data in Figure 4g, which is notably higher than the sample without a corrosion inhibitor (77.6°). This suggests that the incorporation of HMTA resulted in the establishment of an efficacious hydrophobic coating on the metallic surface. This coating alters the surface energy of the metal, thereby reducing its corrosion by the corrosive medium.

**Figure 5:**
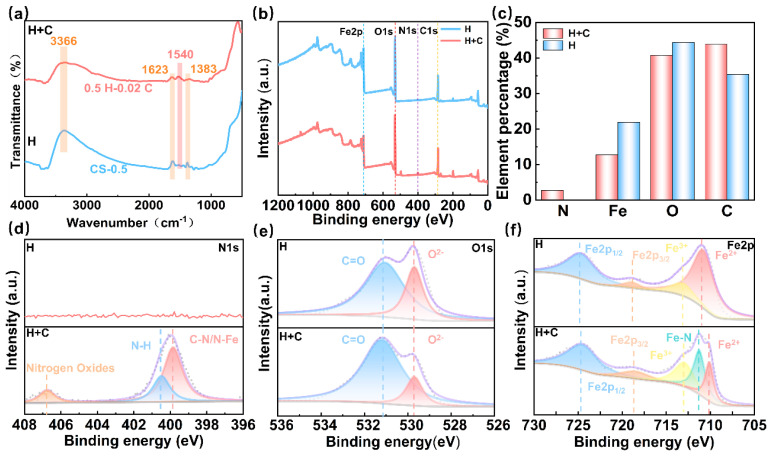
(**a**) Fourier infrared spectroscopy; (**b**) XPS scanning spectrum (0.5 M H; 0.5 M H + 0.02 C); (**c**) atomic fractions of different samples; and (**d**) N 1s spectra, (**e**) O 1s spectra, and (**f**) Fe 2p spectra.

For the sake of analyzing the protective mechanism of HMTA and its effect on the surface elements of Q235 steel, FTIR and XPS were used for surface chemical composition testing. Figure 5a shows the FTIR spectra of the carbon steel sample after immersing in 0.5 M H and 0.5 M H + 0.02 M C electrolyte solutions for 6 h. The same few peaks appear in the spectra of both samples; the peak at 3366 cm^−1^ is associated with the O-H bond’s stretching vibration, while the peak at 1623 cm^−1^ likely corresponds to the C=C bond’s stretching vibration. Additionally, the peak at 1383 cm^−1^ is linked to the bending vibration of the C-H bond [30,31]. Compared to the sample without additives, the H+C spectra show a peak at 1540 cm^−1^ that corresponds to both the N-H bending vibration and the C-N stretching vibration, which means the corrosion inhibitor molecules generate new chemical bonds on the metal surface. This suggests that the corrosion inhibitor is effectively adsorbed onto the metal surface, forming a stable chemical bond.

FTIR spectroscopy has provided evidence that HMTA inhibits etching by generating a sheltered film on the Q235 surface, but the process of film formation is still poorly understood. Therefore, further XPS analysis was performed, and the experimental results are displayed in Figure 5b–f. Figure 5b,c, respectively, show and summarize the full spectra and atomic percentage fractions of different elements on the surface of Q235 samples after immersion in various electrolytes. The results show that all samples have C 1s (284.8 eV), O 1s (531.8 eV), and Fe 2p (711.8 eV) peaks, while an additional N 1s (400.0 eV) peak appears on the sample surface after immersion in the HCl + HMTA electrolyte [32,33]. Further analysis of the atomic percentages of various elements shows a significant decrease in the proportions of Fe and O. However, the share of element C rises, and the atomic share of element N rises even more from 0% to 2.72%. The above analyses indicate that HMTA molecules modify the chemical composition of the Q235 sample surface.

As illustrated in Figure 5d–f and Figure S17, the presence of different bonds in the Q235 samples further supports the adsorption of HMTA molecules on the Q235 sample. More specifically, the detailed spectra of C 1s consist of three peaks associated with C-C/C=C (284.8 eV), C-O (286.1 eV), and C=O (288.4 eV) bonds. The intensity of the C 1s peak is increased compared to the blank sample, likely indicating the adsorption of HMTA molecules with a multi-nitrogen heterocyclic structure on the surface of Q235 steel. Each ring structure in the HMTA molecule contains six carbon atoms, and the addition of HMTA further increases the C concentration on the sample surface [34,35]. As illustrated in Figure 5d, the N 1s spectrum can be divided into three peaks: C-N/N-Fe (399.8 eV), N-H (400.4 eV), and NOx (406.6 eV). The existence of the N-Fe bond indicates that N atoms in the corrosion inhibitor successfully adsorb onto the metal surface. Regarding O 1s, it can be decomposed into two peaks: O^2−^ (529.6 eV) and C=O (531.8 eV). HMTA reduces the O^2-^ peak intensity, proving it effectively protects Q235 steel by isolating the substrate and minimizing oxidation [36].

Finally, two samples both show Fe^2+^ (710.8 eV/710.2 eV) and Fe^3+^ (712.8 eV) and two satellite peaks, Fe 2p_1/2_ (718.8 eV) and Fe 2p_3/2_ (724.6 eV). Notably, sample H+C shows additional Fe-N (711.3 eV); this bond suggests that the lone pair electrons of nitrogen atoms in HMTA form a coordination bond with iron atoms on the sample surface and develop an adsorption layer [37]. The presence of these bonds proves that the HMTA molecules are successfully bonded to the carbon steel, rather than merely leaving residual elements after immersion treatment. FTIR and XPS analyses clearly reveal the role of HMTA molecules in forming a protective layer on the metal surface and their impact on the surface chemistry of Q235, which promotes a clearer understanding of the protective mechanism of the corrosion inhibitor.

To understand how HMTA influences corrosion kinetics, a range of electrochemical tests was used to investigate the kinetic parameters of various samples. The kinetic study of metal corrosion is carried out on a carbon steel substrate using kinetic potential polarization measurements. The corrosion kinetic parameters, specifically the corrosion potential (*E_corr_*) and corrosion current density *(I_corr_*), were obtained through the fitting of the polarization curves using Tafel extrapolation. Accordingly, the potential difference (∆) and the coverage values for different concentrations of HMTA on the Q235 substrate are calculated. Their formula is as follows [38,39]:

(1) $$∆={\;E}_{corr\;(undeformed)}\;-\;\;E_{corr\;(deformed)}$$

(2) $$\theta \;=\;1\;-\;\frac{{I^{\text{’}}}_{corr}}{{I^{0}}_{corr}}$$

**Figure 6:**
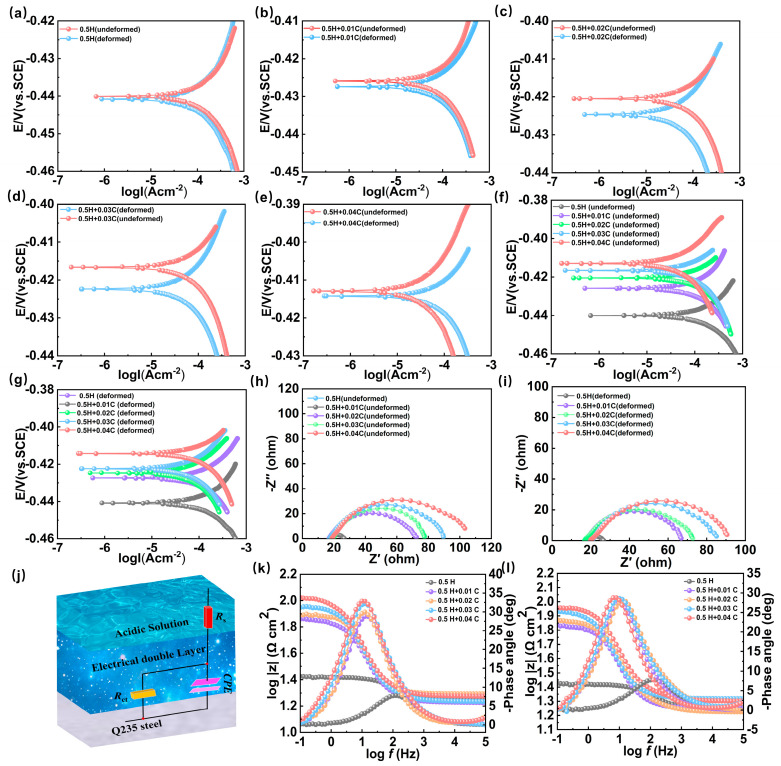
(**a**–**e**) Polarization curves of the deformed and undeformed regions of Q235 when the electrolyte is 0.5 M hydrochloric acid and 0 M, 0.01 M, 0.02 M, 0.03 M, and 0.04 M HMTA are added, respectively; plot of metal polarization curves in the (**f**) undeformed zone and (**g**) deformed zone with different concentrations of corrosion inhibitors; Nyquist plots of (**h**) undeformed metals and (**i**) deformed metals at different corrosion inhibitor concentrations; (**j**) equivalent circuit used; and Bode plots of (**k**) undeformed metals and (**l**) deformed metals at different corrosion inhibitor contents.

Firstly, from the basic principle of the electrolytic method for the reappearance of the VIN, the relationship between the revealing effect of the electrolytic method and the potential difference is analyzed. According to what is described by Figure 6a–e and Table 2, with the addition of HMTA, the potentiometric difference between the metal deformation area and the undeformed area shows a trend of rising first and then falling, and reaches the maximum value at 0.03 M. Further analysis of the causes for the potential difference reveals that the exterior of the undeformed region is relatively intact, and HMTA forms a uniform and stable protective layer on its surface, which effectively slows down the corrosion reaction [40,41]. In contrast, there are concentrated stresses inside the deformation region and defects on the Q235 surface, which may result in a less uniform protective layer of corrosion inhibitor. More specifically, when the carbon steel deformation area lacks protection, the current forms a microcell on the metal surface, and galvanic coupling corrosion occurs, which leads to more severe localized corrosion at the site of the electrochemical reaction. As a result, the potential difference increases with increasing HMTA concentrations, and the corrosion contrast becomes more intense. However, when the corrosion inhibitor concentration reaches 0.04 M, the potential difference Δ begins to decrease, which may be due to the insufficient relative saturation of the corrosion inhibitor concentration. At this time, the HMTA molecules can also generate a dense preservative film on the deformed substrate, leading to a decline in the potentiometric disparity and a weaker contrast effect between the two regions.

According to what is described by Figure 6f,g and Table 2, as opposed to the blank solution, the *E_corr_* of both deformed and undeformed areas are shifted in the positive direction after the introduction of the corrosion inhibitor. Meanwhile, the surface coverage value of HMTA on the metal substrate grows with the rise in HMTA concentration. On the contrary, the *I_corr_* decreases with increasing corrosion inhibitor concentration. This trend indicates that the HMTA molecules are successfully adsorbed on the metal surface and successfully exert an anticorrosion effect during the corrosive progression. Figure 6h,i show Nyquist diagrams of the Q235 sample with various proportions of HMTA added to the deformed and undeformed regions, respectively. The impedance behavior of the Q235 electrode can be used to fit its circuit with Figure 6j. Among them, *R_s_* represents the solution resistance, and *R_ct_* stands for the charge-transfer resistance [42]. Combined with Table 3, it can be observed that with the rising proportion of HMTA, the *η* value and impedance arc diameter also increase. This suggests that the anticorrosive agent molecules are attached to the surface of carbon steel, which hinders the charge-transfer progression and reduces the corrosion speed [43,44].

From the Bode plot (Figure 6k,l), it is evident that with the increase in the proportion of anticorrosive agent, the mid-frequency region exhibits higher phase angle values. This is because of the increasing density of the adsorbed layer, and the corrosion inhibitor film significantly obstructs the active positions on the Q235 surface, thereby suppressing the corrosion rate. As HMTA content increases, the impedance modulus also gradually increases, indicating that the introduction of an anticorrosive agent significantly improves the electrochemical performance of the sample, which enhances its capacitive characteristics and inhibits the corrosion reaction [45]. Electrochemical analyses show that the potential difference (Δ) increases significantly after the addition of HMTA, which fully reveals the key role played by the protective effect of HMTA in enhancing the VIN reappearance effect. In addition, the characterization visualizes the additive effect by means of electrochemical parameters, which helps to further understand the influence of various proportions of HMTA on the VIN reproduction effect.

**Figure 7:**
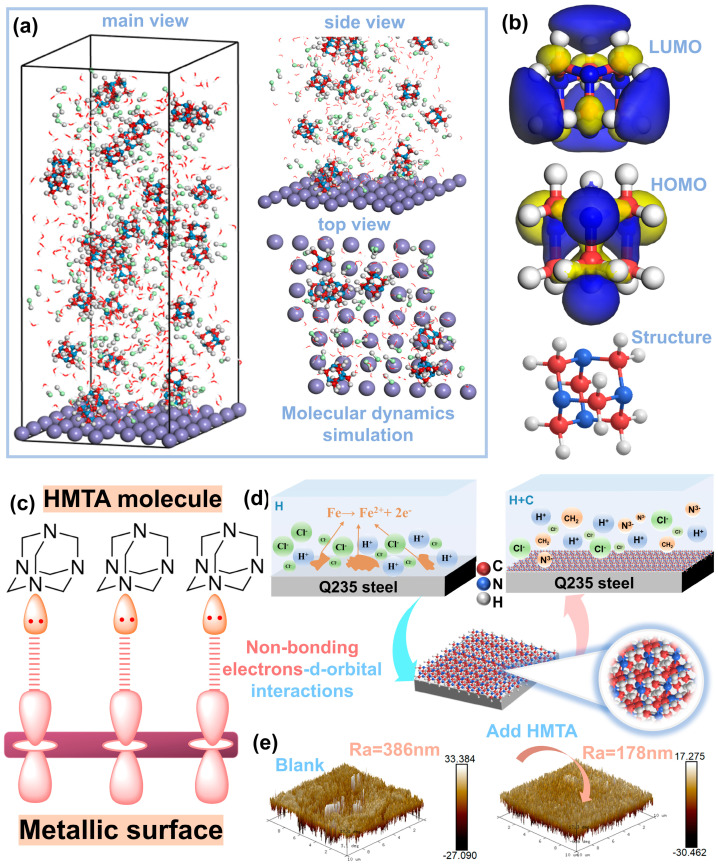
(**a**) Equilibrium configuration; (**b**) optimized conformations of HMTA molecules, LUMO and HOMO; (**c**) schematic diagram of N atom adsorption; (**d**) schematic diagram of HMTA corrosion inhibition process; and (**e**) AFM morphology of Q235 after immersion in 0.5 M H (blank) and 0.5 M H + 0.02 M C (add HMTA) electrolyte 6H, respectively.

With the aim of further revealing the connection between the studied corrosion inhibitor molecules and the carbon steel surface, molecular dynamics simulations and density functional theory calculations were conducted in this study. Figure 7a illustrates the adsorption configuration of HMTA on the Fe (110) surface, and multiple HMTA molecules can be detected as being attached to the carbon steel. HMTA has a cage-like structure with multiple six-membered rings connected by nitrogen atoms, and its stable ring structure enables it to be stably attached to the metallic surface. More specifically, the nitrogen atoms in the corrosion inhibitor molecules establish coordination bonds with the iron atoms to cover the active sites at the metal interface, forming a barrier on the carbon steel surfaces and thus effectively reducing the erosion of H^+^ and Cl^−^ [46,47]. The adsorption energy of HMTA on the Fe (110) surface can be calculated by the following equation [48].

(3) $$E_{ads}={\;E}_{tot}\;-\;(E_{surf+solu}+E_{inh+solu})$$

Here, *E_tot_* represents the total energy; *E_solu_* is the solution energy; *E_surf+solu_* is the total energy without HMTA; and *E_inh+solu_* represents the sum of the corrosion inhibitor and solution energy. Broadly speaking, the more negative the value of E_ads_, the stronger the interaction between the adsorbent and adsorbate. From the calculations, we obtain the value of the adsorption energy as −64.8734 kJ/mol, which indicates that the model system is stable and HMTA has a high adsorption capacity on the Fe (110) surface [49].

Classically, the HOMO orbital is related to the electron-donating ability of the inhibitor molecule, and a larger value indicates a greater electron-giving ability. On the contrary, the LUMO orbital is associated with the electron-accepting capability of the molecule, and the smaller the value is, the greater the electron-absorbing ability of the molecule [50]. The energy gap value (ΔE = ELUMO − EHOMO) is commonly employed to assess the corrosion protection performance of corrosion inhibitors. The Δ*E* value of HMTA is 6.008 eV, indicating an excellent corrosion protection performance [39]. By further analyzing the distribution positions of HOMO and LUMO in Figure 7b, it is found that the electron cloud is mainly distributed on the N and H atoms. As a result, these positions in HMTA are preferably attached to the surface of carbon steel. In detail, N atoms may be partially negatively charged due to their high electronegativity, which promotes their adsorption with iron cations on the carbon steel surface (Figure 7c). In contrast, metal atoms on the surface of Q235 are usually positively charged due to the lack of saturated coordination sites on the surface or because of the formation of an iron oxide layer by oxidation, which allows them to be attracted to the negatively charged portion of the HMTA molecule.

From the schematic diagram (Figure 7d), it can be observed that the HMTA molecules create a shielding layer on the carbon steel surface and slow down the corrosion of hydrochloric acid, in contrast to the blank sample. Briefly, the presence of electrons in the HOMO orbitals of HMTA can interact with the acceptor orbitals over the steel surface, and the LUMO orbitals can accept the charge from the electrons on the metal surface, which leads to the establishment of stable chemical coordination bonds and an adsorption layer on the Q235 surface. It effectively isolates the etching medium from eroding the surface of Q235 [51]. Furthermore, the AFM test in Figure 7e is also consistent with the MD results, and the average coarseness of the metal surface after the addition of the HMTA decreases from 386 nm to 178 nm, which is a clear effect of corrosion inhibition [52]. Through DFT calculations and MD simulations, we have corroborated and analyzed the mechanism of HMTA’s role in modulating the reaction kinetics from a theoretical point of view. The experimental results show that HMTA molecules generate an adsorption film through establishing chemical coordination bonds on the carbon steel surface. This dramatically changes carbon steel electrode surface characteristics while significantly reducing the activity of the electrode towards the corrosion reaction. Thereby, it has a crucial function in controlling the rate of reaction kinetics during the corrosion process.

## 4. Conclusions

This study demonstrates a simple and effective electrochemical etching method that uses 0.5 M HCl with 0.02–0.03 M HMTA at 6 V. It achieves high-contrast VIN reappearance on Q235 steel within 3–4 min and outperforms conventional techniques in speed by more than five times. The theoretical and experimental results reveal a dual mechanism of HMTA: (1) the formation of the N-Fe bound protective layer reduces the roughness of the metal surface in the undeformed region (about 54%) and contributes to the frame number character region with a higher selective anodic oxidation reaction; (2) the corrosion potential difference (ΔE_max_ = 6 mV) was selectively enhanced between deformed and intact regions, as validated by electrochemical characterization. The precision and reproducibility of this method highlight the immediate forensic utility for VIN recovery and have potential applications in firearms, precision instruments, and jewelry serial number detection. The future work will address its adaptation for corrosion-resistant alloys in order to broaden the practical scope.

## Figures and Tables

**Table 1:** Experimental data from the electrolysis method when different concentrations of hexamethylenetetramine (HMTA) were added to the electrolyte of 0.5 M HCl at a voltage of 6 V.

Reagent (M)	Current (A)	Average Time (S)	Experimental Results
0	0.25~0.53	-	Failure to reappear
0.01	0.29~0.45	174	Recognizable but blurry
0.02	0.29~0.47	198	Clear contrast is obvious
0.03	0.33~0.57	200	Clear contrast is obvious
0.04	0.33~0.59	207	Recognizable but blurry

**Table 2:** Electrochemical fitting parameters of mild steel electrode in corrosive medium with different contents of HMTA + 0.5 M HCl.

Metallic Condition	Concentration (M)	−*E*_corr_ (mV)	*I*_corr_ × 10^−3^ (A/cm^−2^)	Δ (mV)	θ
Undeformed	0	439	0.664	1	-
	0.01	426	0.506	2	0.237
	0.02	420	0.293	5	0.558
	0.03	416	0.196	6	0.704
	0.04	412	0.165	2	0.751
Deformed	0	440	0.887	1	-
	0.01	427	0.56	2	0.368
	0.02	425	0.493	5	0.444
	0.03	422	0.332	6	0.625
	0.04	414	0.278	2	0.686

**Table 3:** Fitted parameters of electrochemical impedance of mild steel electrode in corrosive medium with different concentrations of HMTA added to 0.5 M HCl.

Metallic Condition	Concentration (M)	*R*_s_ (Ω cm^2^)	*R_c_*_t_ (Ω cm^2^)	*Y*_0_ × 10^−6^ (S s^n^ cm^−2^)	n	*η* (%)
undeformed	0	17.97	8.37	856.73	0.7908	-
	0.01	16.97	55.41	799.73	0.7997	84.89
	0.02	19.73	59.29	765.87	0.8563	85.88
	0.03	17.35	74.07	792.55	0.7925	88.69
	0.04	18.94	87.62	780.98	0.7809	90.44
deformed	0	17.98	8.29	1322	0.7967	-
	0.01	17.88	50.15	1198	0.8214	83.46
	0.02	17	57.61	1148	0.7628	85.61
	0.03	20.93	64.61	1159	0.8235	87.16
	0.04	20.34	73.12	1086	0.7664	88.66

## Data Availability

All the relevant data are included in this published article.

## Supplementary Materials

The following supporting information can be downloaded at: https://www.mdpi.com/article/10.3390/mi16050578/s1, Figure S1. Results of electrolysis when the electrolyte is 0.3 M HCl. Table S1. Electrolysis data with 0.3 M HCl electrolyte. Figure S2. Results of electrolysis when the electrolyte is 0.3 M HCl + 0.01 M HMTA. Table S2. Electrolysis data with 0.3 M HCl + 0.01 M HMTA electrolyte. Figure S3. Results of electrolysis when the electrolyte is 0.3 M HCl + 0.02 M HMTA. Table S3. Electrolysis data with 0.3 M HCl + 0.02 M HMTA electrolyte. Figure S4. Results of electrolysis when the electrolyte is 0.3 M HCl + 0.03 M HMTA. Table S4. Electrolysis data with 0.3 M HCl + 0.03 M HMTA electrolyte. Figure S5. Results of electrolysis when the electrolyte is 0.3 M HCl + 0.04 M HMTA. Table S5. Electrolysis data with 0.3 M HCl + 0.04 M HMTA electrolyte. Figure S6. Results of electrolysis when the electrolyte is 0.5 M HCl. Table S6. Electrolysis data with 0.5 M HCl electrolyte. Figure S7. Results of electrolysis when the electrolyte is 0.5 M HCl + 0.01 M HMTA. Table S7. Electrolysis data with 0.5 M HCl + 0.01 M HMTA electrolyte. Figure S8. Results of electrolysis when the electrolyte is 0.5 M HCl + 0.02 M HMTA. Table S8. Electrolysis data with 0.5 M HCl + 0.02 M HMTA electrolyte. Figure S9. Results of electrolysis when the electrolyte is 0.5 M HCl + 0.03 M HMTA. Table S9. Electrolysis data with 0.5 M HCl + 0.03 M HMTA electrolyte. Figure S10. Results of electrolysis when the electrolyte is 0.5 M HCl + 0.04 M HMTA. Table S10. Electrolysis data with 0.5 M HCl + 0.04 M HMTA electrolyte. Figure S11. Results of electrolysis when the electrolyte is 0.7 M HCl. Table S11. Electrolysis data with 0.7 M HCl electrolyte. Figure S12. Results of electrolysis when the electrolyte is 0.7 M HCl + 0.01 M HMTA. Table S12. Electrolysis data with 0.7 M HCl + 0.01 M HMTA electrolyte. Figure S13. Results of electrolysis when the electrolyte is 0.7 M HCl + 0.02 M HMTA. Table S13. Electrolysis data with 0.7 M HCl + 0.02 M HMTA electrolyte. Figure S14. Results of electrolysis when the electrolyte is 0.7 M HCl + 0.03 M HMTA. Table S14. Electrolysis data with 0.7 M HCl + 0.03 M HMTA electrolyte. Figure S15. Results of electrolysis when the electrolyte is 0.7 M HCl + 0.04 M HMTA. Table S15. Electrolysis data with 0.7 M HCl + 0.04 M HMTA electrolyte. Figure S16. When the voltage is 6V and the electrolyte is 0.5H + 0.03C, the results are reproduced by the electrolysis method. Figure S17. C 1s spectra of Q235 after being immersed in 0.5 M H and 0.5 M H + 0.02 C electrolytes for 6h. Figure S18. Experimental results of simulated applications. Figure S19. Electrolysis results at 6 V and 0.5 M HCl + various HMTA concentrations. Table S16. Experimental data of the electrolysis method when different concentrations of HMTA are added to the electrolyte of 0.5 M HCl at a voltage of 6V. Figure S20. Electrolysis results at 6 V and 0.5 M HCl + various HMTA concentrations. Table S17. Experimental data of the electrolysis method when different concentrations of HMTA are added to the electrolyte of 0.5 M HCl at a voltage of 6V. Table S18. The sensitivity and efficacy of four common chemical etching reagents in restoring the latent stamped characters on the metal surface (INDIAN INSTITUTE OF METALS, 2020, 73,1867-1878).
