# Supplementary material for: Modulating Reaction Kinetics Using an Electrolytic Method to Achieve Efficient Vehicle Identification Number Reappearance
Source: Micromachines (Basel). 2025 May 15;16(5):578. doi: 10.3390/mi16050578 (PMC12114170; doi:10.3390/mi16050578)
Supplement: Supplementary file 1 [file micromachines-16-00578-s001.zip › micromachines-3614906-supplementary.pdf]

Supplementary Materials

Modulating Reaction Kinetics Using an Electrolytic Method to Achieve Efficient Vehicle Identification Number Reappearance

Jintao Wang [a], Xiaoshun Zhang [a, b*], Mengfan Chen [c], Xihao Zhang [d], Zhongliang Zhang [a, b]

[a] Criminal Investigation Police University of China, Shenyang 116049, Liaoning, China

[b] Key Laboratory of Impression Evidence Examination and Identification Technology, Ministry of Public Security ,Shenyang 110035, China

[c] School of Chemistry and Life Science, Anshan Normal University, Anshan 114007, Liaoning, China

[d] Institute of Metal Research, Chinese Academy of Sciences, Shenyang 110016, Liaoning, China

* Corresponding author.
E-mail address: ddzhangxiaoshun@163.com

To optimize the electrolysis conditions for VIN recovery, we designed systematic experiments. The concentrations of hydrochloric acid were 0.3 M, 0.5 M, and 0.7 M. Different concentrations of corrosion inhibitors were added to the hydrochloric acid electrolyte system (0 M, 0.01 M, 0.02 M, 0.03 M, and 0.04 M). The specific experimental results are as follows.

1. The concentration of hydrochloric acid is 0.3 M; the concentrations of HMTA are 0 M, 0.01 M, 0.02 M, 0.03 M, and 0.04 M; and the electrolytic voltages in each set of graphs are 4V, 6V, 8V, and 10V from top to bottom, respectively.

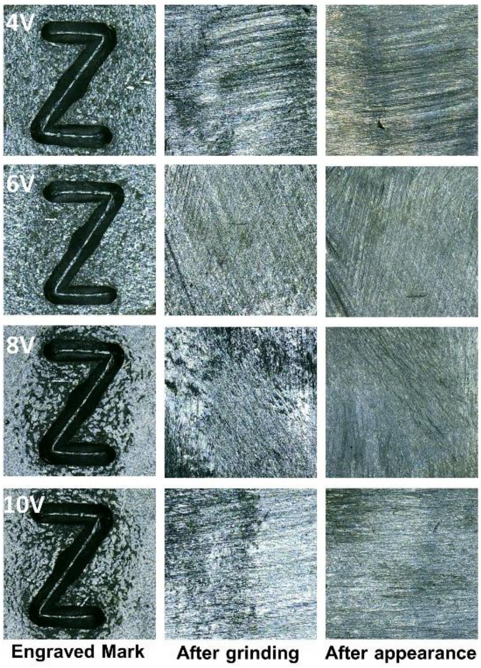

Figure S1. Results of electrolysis when the electrolyte is 0.3 M HCl.

Table S1. Electrolysis data with 0.3 M HCl electrolyte.

Voltage (V)	Current (A)	Times（Min）	Appearance	Result
4	0.11~0.20	-	There are tiny bubbles coming out of the cotton sleeve, and the color of the cotton sleeve is greenish in the contact area with the carbon steel.	Failure to appear
6	0.19~0.33	-	The number of air bubbles on the cotton sleeve increases, the color of the contact area with the metal changes from green to yellow, and the steel plate is slightly hot.	Failure to appear
8	0.19~0.36	-	A large number of air bubbles appear on the cotton cover and burst immediately; the cotton cover begins to dissolve.	Failure to appear
10	0.22~0.36	-	A large amount of black material is left on the carbon steel, the carbon steel heats up badly, and the power warning light blinks.	Failure to appear

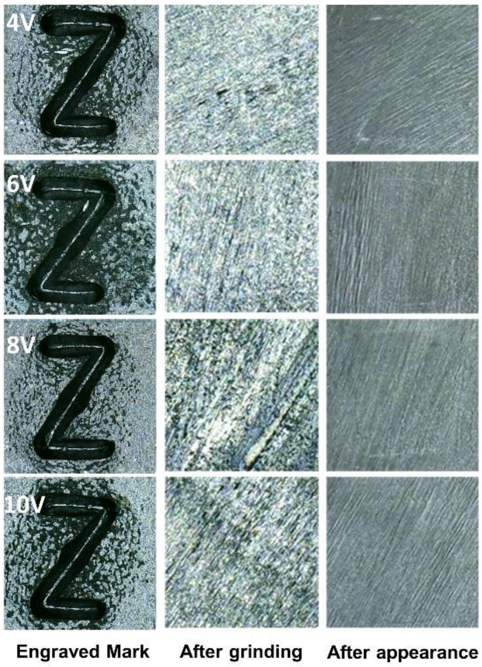

Figure S2. Results of electrolysis when the electrolyte is 0.3 M HCl + 0.01 M HMTA.

Table S2. Electrolysis data with 0.3 M HCl + 0.01 M HMTA electrolyte.

Voltage (V)	Current (A)	Times（Min）	Appearance	Result
4	0.10~0.21	9.10	Air bubbles intermittently form on the cotton sleeve surface and promptly rupture.	Character recovery marks can be largely distinguished with poor contrast.
6	0.18~0.31	8.37	The number of air bubbles on the cotton sleeve increases, and the area of contact between the sleeve and the carbon steel turns blue.	Character recovery marks can be largely distinguished with poor contrast.
8	0.20~0.36	7.10	There is no significant change in the number of air bubbles on the cotton cover, and the color of the cotton cover changes from green to black.	Character recovery marks can be largely distinguished with poor contrast.
10	0.22~0.38	6.02	The head of the cotton sleeve is blackened, the tail is yellowed, and the number of air bubbles increases.	Only fuzzy letter latent images are seen (fail).

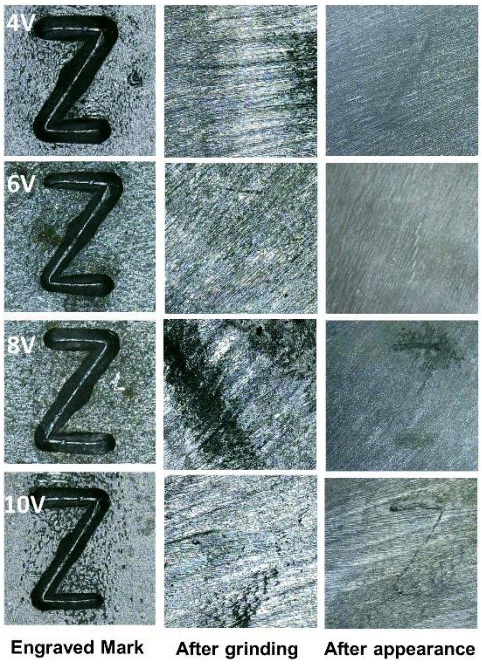

Figure S3. Results of electrolysis when the electrolyte is 0.3 M HCl + 0.02 M HMTA.

Table S3. Electrolysis data with 0.3 M HCl + 0.02 M HMTA electrolyte.

Voltage (V)	Current (A)	Times （Min）	Appearance	Result
4	0.09~0.24	12.46	There are tiny bubbles coming out of the cotton sleeve, and the color of the cotton sleeve is greenish in the contact area with the carbon steel.	Character recovery marks can be largely distinguished with poor contrast.
6	0.13~0.31	8.40	The number of air bubbles on the cotton sleeve increases, the color of the contact area with the metal changes from green to yellow, and the steel plate is slightly hot.	Character recovery marks can be largely distinguished with poor contrast.
8	0.15~0.49	8.05	A large number of bubbles appear on the cotton sleeve and immediately burst, and the cotton sleeve begins to dissolve. Carbon steel is slightly hot.	Character recovery marks can be largely distinguished with poor contrast.
10	0.21~0.45	6.48	There is no significant change in the number of air bubbles on the cotton sleeve, the color of the head of the cotton sleeve turns from green to black, the tail is yellow, and the carbon steel is slightly hot.	Reproduces characters clearly.

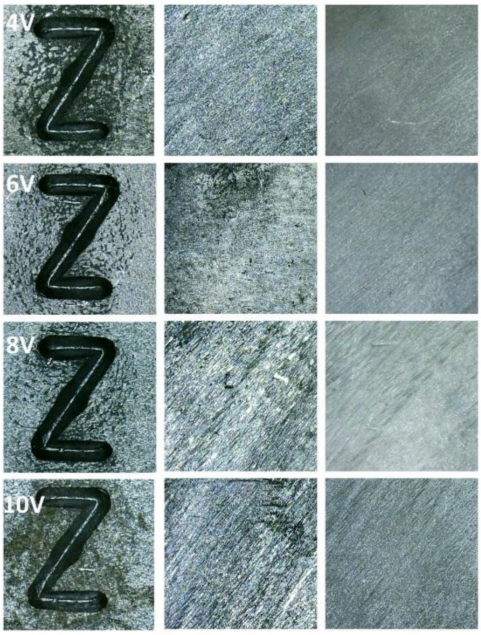

Figure S4. Results of electrolysis when the electrolyte is 0.3 M HCl + 0.03 M HMTA.

Table S4. Electrolysis data with 0.3 M HCl + 0.03 M HMTA electrolyte.

voltage (V)	Current (A)	Times （Min）	Appearance	Result
4	0.06~0.17	8.03	There are tiny bubbles coming out of the cotton sleeve, and the color of the cotton sleeve is greenish in the contact area with the carbon steel.	Character recovery marks can be largely distinguished with poor contrast.
6	0.17~0.29	7.40	The number of air bubbles on the cotton sleeve increases, the contact area between the cotton sleeve and the carbon steel is green, and the carbon steel was slightly hot.	Only fuzzy letter latent images, fail.
8	0.18~0.34	5.48	There is no obvious change in the number of air bubbles on the cotton cover, the color of the cotton cover changes from green to black, and there is no obvious change in the degree of heat generation.	Character recovery marks can be largely distinguished with poor contrast.
10	0.23~0.41	6.57	There is no significant change in the number of air bubbles on the cotton sleeve, the color of the head of the cotton sleeve turns from green to black, the tail is yellow, and the carbon steel is further heated.	Only fuzzy letter latent images, fail.

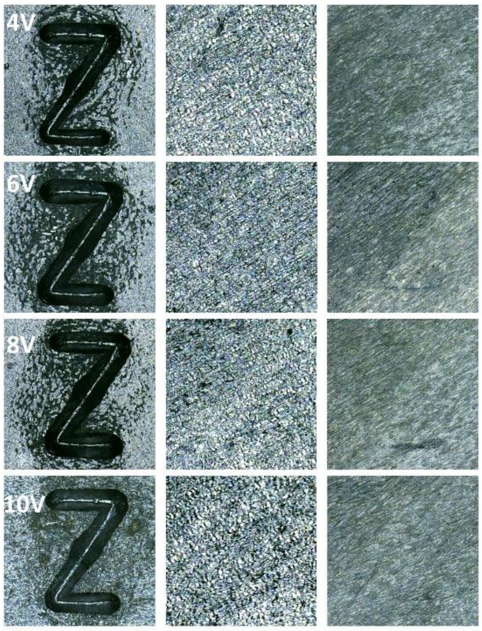

Figure S5. Results of electrolysis when the electrolyte is 0.3 M HCl + 0.04 M HMTA.

Table S5. Electrolysis data with 0.3 M HCl + 0.04 M HMTA electrolyte.

Voltage (V)	Current (A)	Times (Min)	Appearance	Result
4	0.04~0.13	10.31	There are tiny bubbles coming out of the cotton sleeve, and the color of the cotton sleeve is greenish in the contact area with the carbon steel.	Character recovery marks can be largely distinguished with poor contrast.
6	0.16~0.24	8.20	The number of air bubbles on the cotton sleeve increases, the contact area between the cotton sleeve and the carbon steel is green, and the carbon steel is slightly hot.	Character recovery marks can be largely distinguished with poor contrast.
8	0.18~0.34	8.05	There is no obvious change in the number of air bubbles on the cotton cover, the color of the cotton cover changes from green to black, and there is no obvious change in the degree of heat generation.	Only fuzzy letter latent images are seen (fail).
10	0.21~0.39	7.26	There is no significant change in the number of air bubbles on the cotton sleeve, the color of the head of the cotton sleeve turns from green to black, the tail is yellow, and the carbon steel is further heated up.	Only fuzzy letter latent images are seen (fail).

2. The concentration of hydrochloric acid is 0.5 M; the concentration of HMTA is 0 M,

0.01 M, 0.02 M, 0.03 M, and 0.04 M; and the electrolytic voltages in each set of graphs are 4V, 6V, 8V, and 10V, respectively, from top to bottom.

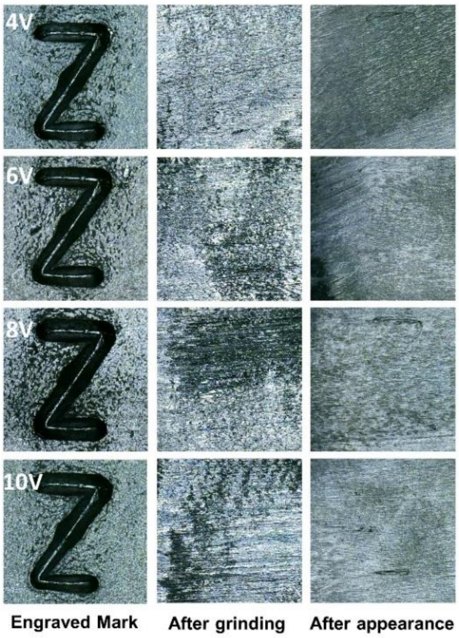

Figure S6. Results of electrolysis when the electrolyte is 0.5 M HCl.

Table S6. Electrolysis data with 0.5 M HCl electrolyte.

Voltage (V)	Current (A)	Times（Min）	Appearance	Result
4	0.2~0.73	3.46	There are tiny bubbles coming out of the cotton sleeve, and the color of the cotton sleeve is greenish in the contact area with the carbon steel.	Only fuzzy letter latent images are seen (fail).
6	0.25~0.93	2.25	The number of air bubbles on the cotton sleeve increases, the color of the contact area with the metal changes from green to yellow, and the steel plate is slightly hot.	Only fuzzy letter latent images are seen (fail).
8	0.53~1.61	1.54	A large number of air bubbles appear on the cotton cover and immediately rupture, the cotton cover begins to dissolve, and a large amount of white smoke with a pungent odor is emitted.	Character recovery marks can be largely distinguished with poor contrast.
10	0.62~1.74	1.40	A large amount of black material is left on the carbon steel, the carbon steel heats up badly, and the power warning light blinks.	Character recovery marks can be largely distinguished with poor contrast.

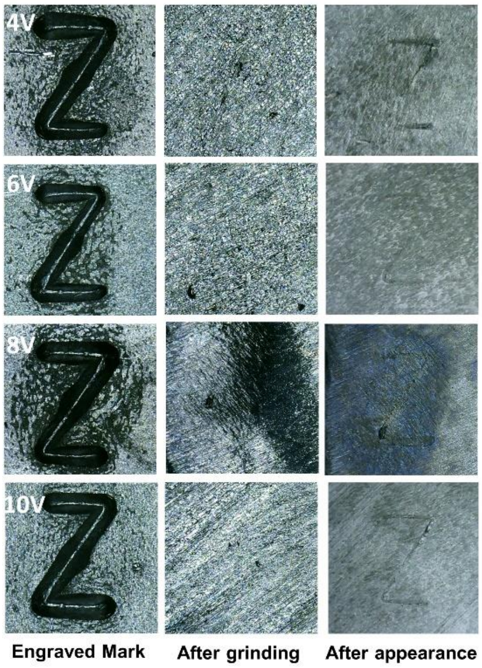

Figure S7. Results of electrolysis when the electrolyte is 0.5 M HCl + 0.01 M HMTA.

Table S7. Electrolysis data with 0.5 M HCl + 0.01 M HMTA electrolyte.

Voltage (V)	Current (A)	Times （Min）	Appearance	Result
4	0.21~0.47	4.29	A large number of tiny air bubbles emerge from the cotton sleeve and quickly rupture, and the end of the cotton sleeve is yellowish in color.	Appearance is obvious, but some strokes are not clear.
6	0.29~0.45	2.58	The number of air bubbles on the cotton sleeve increases, the cotton sleeve is blackened, and the carbon steel is slightly warmed.	Appearance is obvious, but some strokes are not clear.
8	0.32~0.69	2.34	There is no significant change in the number of bubbles, the cotton cover begins to dissolve, leaving a large amount of black material on the steel plate, and the carbon steel heats up severely.	Appearance is obvious, but some strokes are not clear.
10	0.46~1.11	2.17	It is essentially the same as when the voltage is 8V.	Reproduces characters clearly.

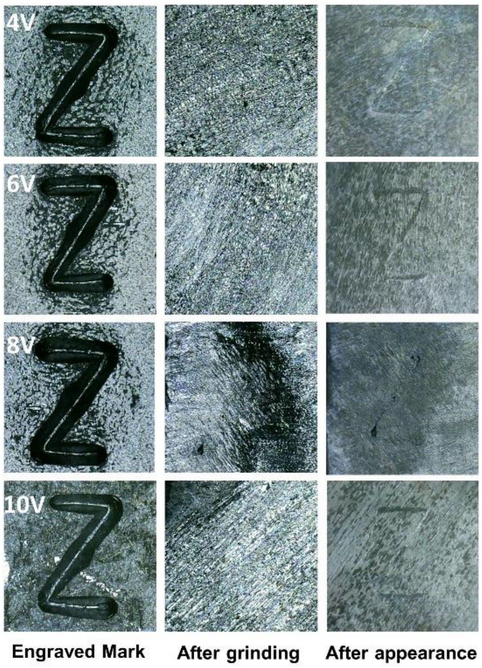

Figure S8. Results of electrolysis when the electrolyte is 0.5 M HCl + 0.02 M HMTA.

Table S8. Electrolysis data with 0.5 M HCl + 0.02 M HMTA electrolyte.

Voltage (V)	Current (A)	Times（Min）	Appearance	Result
4	0.20~0.42	4.25	A large number of tiny air bubbles emerge from the cotton sleeve and quickly rupture, and the end of the cotton sleeve is yellowish in color.	Character recovery marks can be largely distinguished with poor contrast.
6	0.29~0.47	3.21	The number of air bubbles on the cotton sleeve increases, the cotton sleeve is blackened, and the carbon steel is slightly warmed.	Reproduces characters clearly.
8	0.37~0.74	2.47	There is no significant change in the number of bubbles, the cotton cover begins to dissolve, leaving a large amount of black material on the steel plate, and the carbon steel heats up severely.	Character recovery marks can be largely distinguished with poor contrast.
10	0.41~0.95	2.25	It is essentially the same as when the voltage is 8V. Carbon steel heats up further.	Appearance is obvious, but some strokes are not clear.

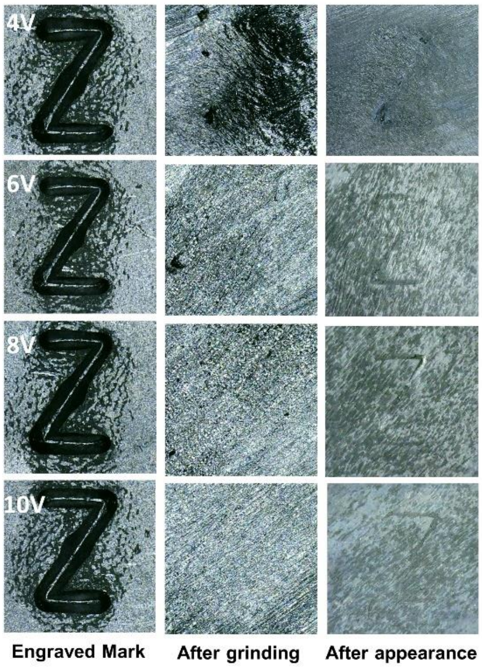

Figure S9. Results of electrolysis when the electrolyte is 0.5 M HCl + 0.03 M HMTA.

Table S9. Electrolysis data with 0.5 M HCl + 0.03 M HMTA electrolyte.

Voltage (V)	Current (A)	Times （Min）	Appearance	Result
4	0.14~0.26	6.10	A large number of tiny air bubbles emerge from the cotton cover and quickly rupture, and the overall color of the cotton cover is yellow.	Character recovery marks can be largely distinguished with poor contrast.
6	0.33~0.57	3.14	The number of air bubbles on the cotton sleeve increases, the cotton sleeve is blackened, and the carbon steel is slightly warmed.	Reproduces characters clearly.
8	0.44~0.83	3.11	There is no significant change in the number of bubbles, the cotton cover begins to dissolve, leaving a large amount of black material on the steel plate, and the carbon steel heats up severely.	Appearance is obvious, but some strokes are not clear.
10	0.48~0.91	2.26	It is essentially the same as when the voltage is 8V. Carbon steel heats up further.	Appearance is obvious, but some strokes are not clear.

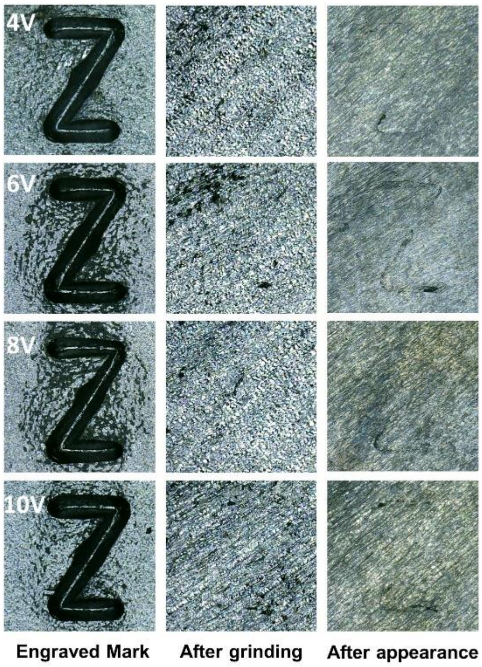

Figure S10. Results of electrolysis when the electrolyte is 0.5 M HCl + 0.04 M HMTA.

Table S10. Electrolysis data with 0.5 M HCl + 0.04 M HMTA electrolyte.

Voltage (V)	Current (A)	Times （Min）	Appearance	Result
4	0.11~0.21	7.03	A large number of tiny air bubbles emerge from the cotton cover and quickly rupture, and the overall color of the cotton cover is yellow.	Character recovery marks can be largely distinguished with poor contrast.
6	0.28~0.46	4.31	The number of air bubbles on the cotton sleeve increases, the cotton sleeve is blackened, and the carbon steel is slightly warmed.	Character recovery marks can be largely distinguished with poor contrast.
8	0.42~0.79	3.41	There is no significant change in the number of bubbles, the cotton cover begins to dissolve, leaving a large amount of black material on the steel plate, and the carbon steel heats up severely.	Only fuzzy letter latent images are seen (fail).
10	0.44~0.86	2.56	It is essentially the same as when the voltage is 8V. Carbon steel heats up further.	Only fuzzy letter latent images are seen (fail).

3. The concentration of hydrochloric acid is 0.7 M; the concentration of HMTA is 0 M, 0.01 M, 0.02 M, 0.03 M, and 0.04 M; and the electrolytic voltages in each set of graphs are 4V, 6V, 8V, and 10V, respectively, from top to bottom.

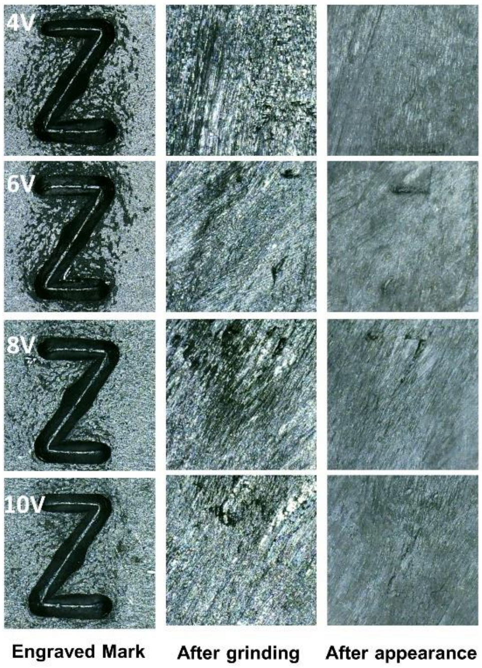

Figure S11. Results of electrolysis when the electrolyte is 0.7 M HCl.

Table S11. Electrolysis data with 0.7 M HCl electrolyte.

Voltage (V)	Current (A)	Times (Min)	Appearance	Result
4	0.73~1.51	2.46	There are tiny bubbles coming out of the cotton sleeve, and the color of the cotton sleeve is greenish in the contact area with the carbon steel.	The corrosion rate is too fast and shows poor results.
6	0.93~1.65	0.54	The number of air bubbles on the cotton sleeve increases, the color of the contact area with the metal changes from green to yellow, and the steel plate is slightly hot.	The corrosion rate is too fast and shows poor results.
8	0.57~1.79	0.34	A large number of air bubbles appear on the cotton cover and immediately rupture, the cotton cover begins to dissolve, and a large amount of white smoke with a pungent odor is emitted.	The corrosion rate is too fast and shows poor results.
10	0.81~1.84	0.22	A large amount of black material is left on the carbon steel, the carbon steel heats up badly, and the power warning light blinks.	The corrosion rate is too fast and shows poor results.

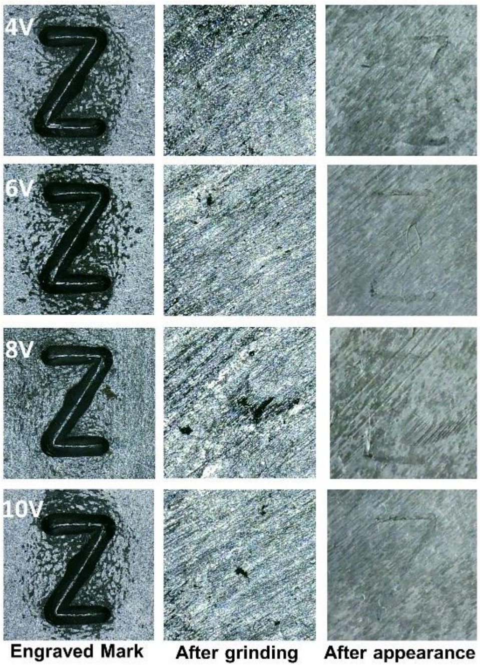

Figure S12. Results of electrolysis when the electrolyte is 0.7 M HCl + 0.01 M HMTA.

Table S12. Electrolysis data with 0.7 M HCl + 0.01 M HMTA electrolyte.

Voltage (V)	Current (A)	Times （Min）	Appearance	Result
4	0.63~1.31	3.24	A large number of air bubbles emerge from the cotton sleeve and quickly burst, the cotton sleeve starts to yellow from the end and quickly turns black, and the carbon steel starts to heat up.	Character recovery marks can be largely distinguished with poor contrast.
6	0.71~1.38	1.17	The number of air bubbles on the cotton sleeve increases further and releases a white, pungent gas.	Reproduces characters clearly.
8	0.79~1.41	1.05	The cotton cover begins to dissolve, the white, pungent gas increases, a large amount of black substance remains on the steel plate, and the power supply's alarm light flashes intermittently, indicating a short circuit condition.	Character recovery marks can be largely distinguished with poor contrast.
10	0.81~1.50	0.58	It is basically the same as when the voltage is 8V, and the power supply is shorted.	Character recovery marks can be largely distinguished with poor contrast.

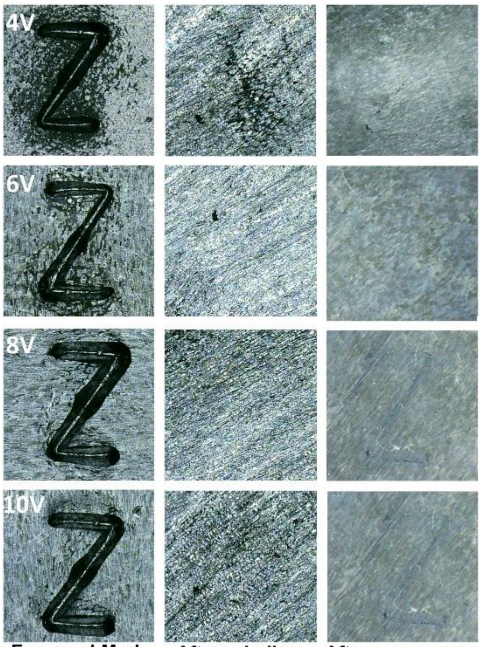

Figure S13. Results of electrolysis when the electrolyte is 0.7 M HCl + 0.02 M HMTA.

Table S13. Electrolysis data with 0.7 M HCl + 0.02 M HMTA electrolyte.

Voltage (V)	Current (A)	Times （Min）	Appearance	Result
4	0.51~0.64	3.47	A large number of air bubbles emerge from the cotton sleeve and quickly burst, the cotton sleeve starts to yellow from the end, and the carbon steel starts to heat up.	Only fuzzy letter latent images are seen (fail).
6	0.61~0.86	2.01	The cotton cover turns black in color, the number of bubbles increases further, and white, pungent gas is emitted. Carbon steel continues to heat up.	Only fuzzy letter latent images are seen (fail).
8	0.72~0.94	1.09	The cotton cover begins to dissolve, leaving a large amount of black material on the steel plate; the plate heats up badly, and the warning light on the power supply flashes.	Character recovery marks can be largely distinguished with poor contrast.
10	0.78~1.13	1.03	It is essentially the same as when the voltage is 8V.	Character recovery marks can be largely distinguished with poor contrast.

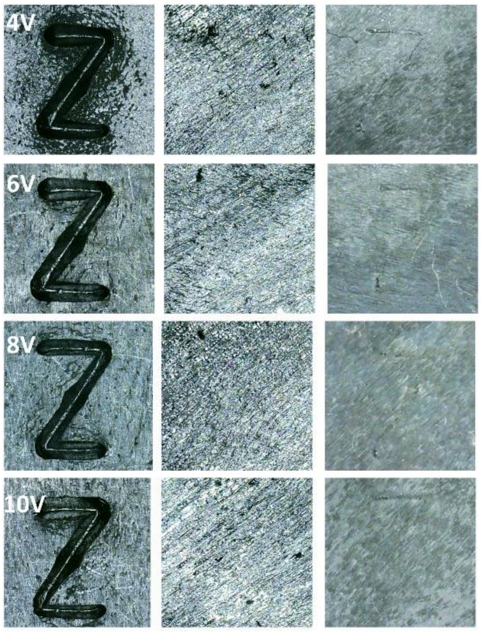

Figure S14. Results of electrolysis when the electrolyte is 0.7 M HCl + 0.03 M HMTA.

Table S14. Electrolysis data with 0.7 M HCl + 0.03 M HMTA electrolyte.

Voltage (V)	Current (A)	Times （Min）	Appearance	Result
4	0.26~0.41	3.02	A large number of air bubbles emerge from the cotton sleeve and quickly burst, the cotton sleeve starts to yellow from the end and quickly turns black, and the carbon steel starts to heat up.	Character recovery marks can be largely distinguished with poor contrast.
6	0.31~0.62	2.16	Bubbles further increase on the cotton cover, white, pungent smoke is emitted, and the carbon steel continues to heat up.	Character recovery marks can be largely distinguished with poor contrast.
8	0.69~1.27	1.48	The cotton cover begins to dissolve, the white, pungent fumes increase, a large amount of black substance remains on the steel plate, and the plate heats up badly.	Character recovery marks can be largely distinguished with poor contrast.
10	0.87~1.41	1.06	It is the same as when the voltage is 8V, the warning light on the power supply is blinking, and the power supply is shorted.	Character recovery marks can be largely distinguished with poor contrast.

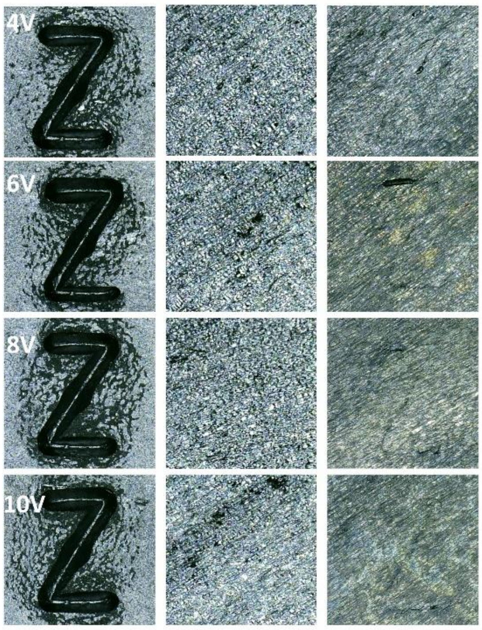

Figure S15. Results of electrolysis when the electrolyte is 0.7 M HCl + 0.04 M HMTA.

Table S15. Electrolysis data with 0.7 M HCl + 0.04 M HMTA electrolyte.

Voltage (V)	Current (A)	Times（Min）	Appearance	Result
4	0.21~0.34	3.13	A large number of air bubbles emerge from the cotton sleeve and quickly burst, the cotton sleeve starts to yellow from the end and quickly turns black, and the carbon steel starts to heat up.	Character recovery marks can be largely distinguished with poor contrast.
6	0.28~0.53	2.46	Bubbles further increase on the cotton cover, white, pungent smoke is emitted, and the carbon steel continues to heat up.	Character recovery marks can be largely distinguished with poor contrast.
8	0.56~1.17	2.01	The cotton cover begins to dissolve, the white, pungent fumes increase, a large amount of black substance remains on the steel plate, and the plate heats up badly.	Character recovery marks can be largely distinguished with poor contrast.
10	0.77~1.29	1.47	It is the same as when the voltage is 8V, the warning light on the power supply is blinking, and the power supply is shorted.	Character recovery marks can be largely distinguished with poor contrast.

Firstly, in terms of electrolyte conditions, the low concentration of 0.3 M

hydrochloric acid leads to limited etching capability and poor overall results. And the 0.7 M hydrochloric acid concentration is too high, which easily causes excessive corrosion. In addition, the high hydrochloric acid concentration will lead to excessive corrosion current and cause serious carbon steel heating and short circuiting of the power supply. There are safety hazards that do not meet the experimental requirements.

Secondly, in terms of voltage, when the voltage is 4 V, the etching rate is low and takes a long time. When the voltage is 8 V and 10 V, the cotton sleeve on the plating pen begins to dissolve and leaves a large amount of black material on the surface of the carbon steel, which prevents real-time observation of the reproduction effect and may lead to excessive etching. In contrast, a voltage of 6 V achieved significant results in terms of reproduction time and safety.

To sum up, this study adopts the method of controlling variables and fixing the voltage at 6V and the concentration of hydrochloric acid at 0.5M and examines the effects of different concentrations of corrosion inhibitors on the reproduction effect of the electrolytic display of the VIN under these experimental conditions.

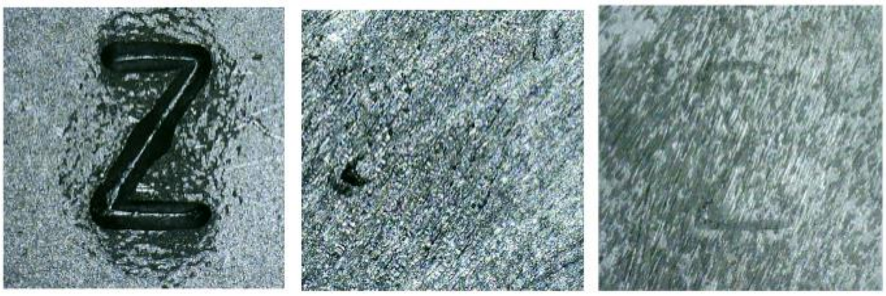

Figure S16. When the voltage is 6V and the electrolyte is 0.5H + 0.03C, the results are reproduced by the electrolysis method.

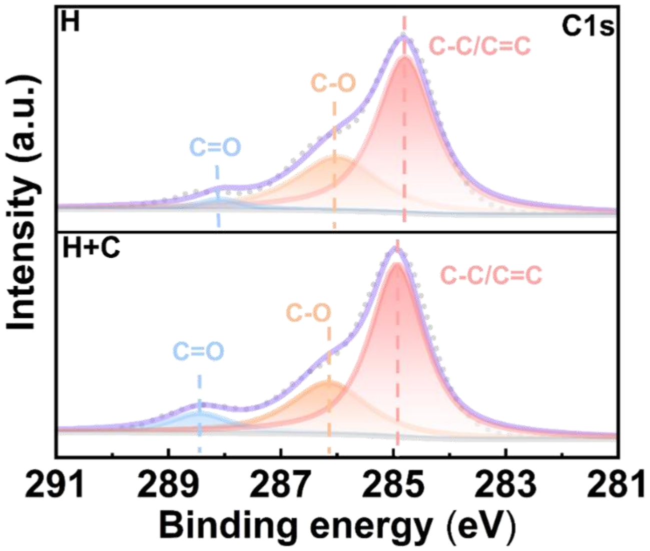

Figure S17. C 1s spectra of Q235 after being immersed in 0.5 M H and 0.5 M H + 0.02 C electrolytes for 6h.

4. Simulation of application experiments

In order to further test the effectiveness of the application of electrolysis for reproducing frame numbers, we engrave a set of serial numbers, including numbers and letters, and eliminate them by simulating the operation of criminals polishing the VIN. On this basis, we implement the reproduction experiment according to the optimal revealing conditions in the experimental results (voltage 6 V; electrolyte 0.5 M HCl + 0.02 M HMTA). The procedure was the same as in Section 2.3.

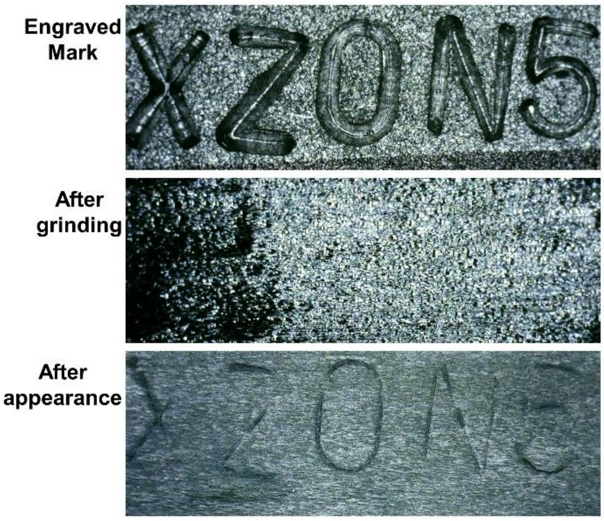

Figure S18. Experimental results of simulated applications.

The experimental results are shown in Fig. S18, and it can be observed that we achieved clear and effective reproducible results to satisfy the working needs of inspection technicians.

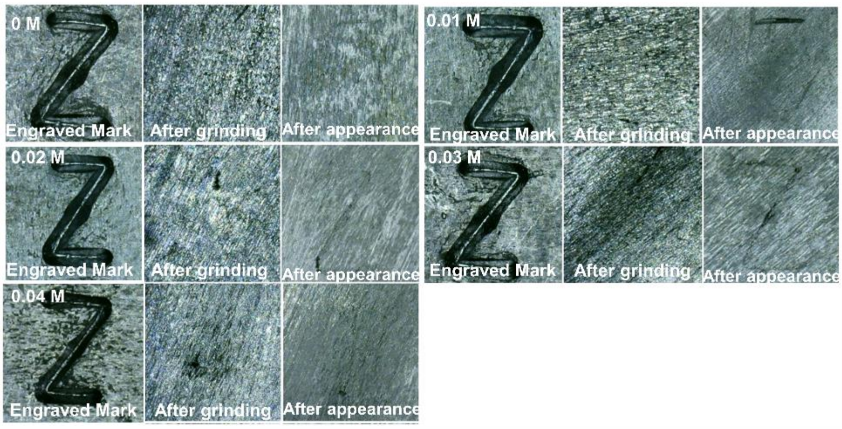

Figure S19. Electrolysis results at 6 V and 0.5 M HCl + various HMTA concentrations

Table S16. Experimental data of the electrolysis method when different concentrations of HMTA are added to the electrolyte of 0.5 M HCl at a voltage of 6V.

Reagent (M)	Current (A)	Time (S)	Experimental results
0	0.27~0.58	-	Failure to reappear
0.01	0.26~0.46	175	Recognizable but blurry
0.02	0.28~0.47	196	Clear contrast is obvious
0.03	0.33~0.57	204	Clear contrast is obvious
0.04	0.33~0.56	210	Recognizable but blurry

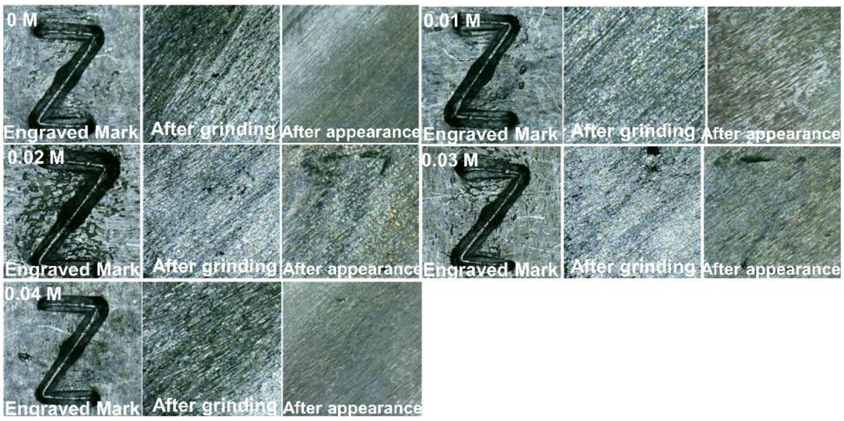

Figure S20. Electrolysis results at 6 V and 0.5 M HCl + various HMTA concentrations.

Table S17. Experimental data of the electrolysis method when different concentrations of HMTA are added to the electrolyte of 0.5 M HCl at a voltage of 6V.

Reagent (M)	Current (A)	Time (S)	Experimental results
0	0.29~0.53	-	Failure to reappear
0.01	0.26~0.45	169	Recognizable but blurry
0.02	0.27~0.47	197	Clear contrast is obvious
0.03	0.29~0.56	203	Clear contrast is obvious
0.04	0.31~0.58	206	Recognizable but blurry

Table S18. The sensitivity and efficacy of four common chemical etching reagents in restoring the latent stamped characters on the metal surface (***INDIAN INSTITUTE OF METALS, 2020, 73,1867-1878***).

Composition	Application Method	Recovery Time	Comments
10 g NaOH + 90 mL H_2O	Continuous swabbing until the marks were fully restored	23-30 min	There was restoration with good contrast.
10% H_3PO_4	Specimen was immersed until the marks were fully recovered	21–23 h	Both contrast and sensitivity were good.
10% NaOH + 10% HNO_3	Alternating swabbing: NaOH (3 min) → HNO_3 (1 min)	58–90 min	The contrasts of the restorations were fair.
60% HCl (37%) + 40% NaOH	Alternating swabbing: HCl (2 min) → NaOH (2 min)	10–15 min	Marks appeared in poor contrast.

We have statistically analyzed the time required for reproducing serial numbers using the chemical etching method in some related studies. The results show that the electrochemical etching method adopted in this study can improve the reproduction speed by more than five times compared with the traditional chemical etching method.
